# Assessing Ecosystem Services Supply-Demand (Mis)Matches for Differential City Management in the Yangtze River Delta Urban Agglomeration

**DOI:** 10.3390/ijerph18158130

**Published:** 2021-07-31

**Authors:** Wenbo Cai, Wei Jiang, Hongyu Du, Ruishan Chen, Yongli Cai

**Affiliations:** 1 School of Design & China Institute for Urban Governance, Shanghai Jiao Tong University, 800 Dongchuan Rd., Minhang District, Shanghai 200240, China; wbcai@rcees.ac.cn (W.C.); chenrsh04@gmail.com (R.C.); 2 Research Center for Eco-Environment Sciences, State Key Laboratory of Urban and Regional Ecology, Chinese Academy of Sciences, Shuangqing Rd. 18, Beijing 100085, China; weijiang@rcees.ac.cn; 3 Institute of Ecology and Sustainable Development, Shanghai Academy of Social Sciences, No.7, Lane 622, Huaihaizhong Road, Huangpu District, Shanghai 200020, China; duhongyu@sass.org.cn

**Keywords:** ecosystem services, supply–demand (mis)matches, urban governance, urbanization, land use, regional sustainable development

## Abstract

With the global increase in population and urban expansion, the simultaneous rise of social demand and degradation of ecosystems is omnipresent, especially in the urban agglomerations of China. In order to manage environmental problems and match ecosystem supply and social demand, these urban agglomerations promoted regional socio-ecological integration but ignored differential city management during the process of integration. Therefore, it is necessary to design a general framework linking ecosystem supply and social demand to differential city management. In addition, in previous studies, ecosystem services supply–demand amount (mis)match assessment was emphasized, but ecosystem services supply–demand type (mis)match assessment was ignored, which may lead to biased decisions. To deal with these problems, this study presented a general ecosystem services framework with six core steps for differential city management and developed a double-indices (amount and type) method to identify ecosystem services supply–demand (mis)matches in an urban agglomeration. This framework and the double-indices method were applied in the case study of the Yangtze River Delta Urban Agglomeration. Ecosystem supply–demand amount and type (mis)match levels and spatial pattern of twenty-six cities were identified. Twenty-six cities in the YRDUA were classified into five kinds of cities with different levels of ES supply–demand (mis)matches for RS, three kinds of cities for PS, and four kinds of cities for CS. Differential city management strategies were designed. Despite its limitations, this study can be a reference to giving insights into ES supply–demand (mis)match assessment and management.

## 1. Introduction

With the global increase in population and urban expansion, the simultaneous rise of social demand and degradation of ecosystems is omnipresent [1,2,3], especially in urban agglomerations of China. China’s reform and opening up accelerated the speed of urbanization, which led to several urban agglomerations such as the Yangtze River Delta Urban Agglomeration (YRDUA), the Pearl River Delta Urban Agglomeration (PRDUA), and the Beijing-Tianjin-Hebei Urban Agglomeration (BTHUA). In these urban agglomerations, the rapid economic development and urban expansion have exacerbated the conflict between construction land and ecological land, leading to serious ecological and environmental problems (e.g., air and water pollution, soil erosion, biodiversity loss) in administrative areas and across administrative areas [4,5]. At the same time, with the improvement of the living standards of urban residents and population growth [3], demands for ecosystem services (ES) have been largely increased in these urban agglomerations, resulting in mismatches of ES supply and demand in cities. To manage environmental problems and to match ecosystem supply and social demand, these urban agglomerations promoted socio-ecological integration and carried out the regional unified city management. However, due to different ecological backgrounds, economic development, and population size, regional unified city management may not be that effective. Therefore, it is necessary to carry out differential city management for cities in these urban agglomerations.

Ecosystem services supply–demand (mis)match assessment may have a great potential in differential land use management [6,7,8]. Ecosystem services (ES) can be defined as direct and indirect contributions to human well-being that originate from ecosystems [9]. ES supply refers to the capacity of a particular area to provide a specific bundle of ecosystem goods and services within a given time period [10]. ES demand was described as the consumption or use of ES in a specific area within a given time period [11] or the preference or expectation level of human society or individuals for the specific attributes of ecosystem services [12]. ES supply–demand (mis)match are defined as the differences in quality or quantity that occur between ES supply and demand in an ecosystem unit or in a specific area [13]. The potential of the different land covers for supplying multiple ES may be the result of biophysical factors or anthropogenic actives, which need certain management to meet people’s demands [14,15]. Therefore, land covers can be classified into three categories, including deficit, stable, and surplus areas of ES [14,15]. Based on ES supply–demand (mis)match assessment with land covers of a city, the differences in ES supply and demand among cities can be identified, and thus cities can be classified. Then, differential city management countermeasures may be proposed by combining an influential factors analysis.

It is necessary to establish comparable methods for ES supply assessment and ES demand assessment. Previous studies mostly focused on the ES supply-side assessment [16,17,18]. Due to the growth of social demand for ES, scholars began to pay attention to ES demand assessment [12,19,20]. In recent studies, comparable ES supply–demand assessment methods have appeared [21,22]. ES supply–demand assessments can be performed by participatory methods [23,24], modeling [25,26], and mapping [27,28]. Modeling was a good choice for quantitative assessments in a data-rich region [25,26], while participatory methods have succeeded in ES supply–demand studies with a combination of mapping methods in a data-scarce region [6,29]. For example, Burkhard et al. [10] developed an ES supply–demand land use matrix based on the participatory mapping. Such a method linking the ES supply–demand assessment to land cover will promote land use management at a regional scale [6,10,11]. Successful cases can be found in regional-scale studies, e.g., assessment of recreational service supply and demand in the Basque Country in Spain [30], and ES supply–demand amount indices for the spatiotemporal analysis at watershed scale in China [31]. Both ES supply and demand can be assessed and visualized by relative comparable units or rates [12]. In addition, in previous studies, the ES supply–demand amount (mis)match assessment was emphasized, but the ES supply–demand type (mis)match assessment was ignored, which may lead to biased decisions [22,32,33,34]. However, the ES supply–demand amount (mis)match assessment could only show the one-side information of the overall status ES supply and demand of a study area, while the ES supply–demand type (mis)match assessment could provide the other-side information in decision making [15,35]. Therefore, the overall assessment should be performed by combing ES supply–demand amount and type match. However, up until present, few studies addressed on both amount and types of ES supply–demand matches for decision making.

The Yangtze River Delta Urban Agglomeration (YRDUA) has large spatial heterogeneity in the ecological background of northern and southern areas. It made great progress in rapid urbanization and economic growth during the past forty years, causing environmental problems such as air pollution, water pollution, and arable land loss, thus threatening human well-being and regional sustainable development [15,36,37,38]. As a typical area of large natural heterogeneity and high intensity of development, YRDUA was selected as the study area for ES supply–demand matches assessment. Taking the YRDUA as an example, the objectives of this study are to (1) present an ES framework for differential city management in an urban agglomeration, (2) develop a double-indices method for identification of ES amount and type (mis)matches in a city, and (3) classify cities in the YRDUA for differential city management based on the double-indices method.

## 2. Methodology

**Figure 1:**
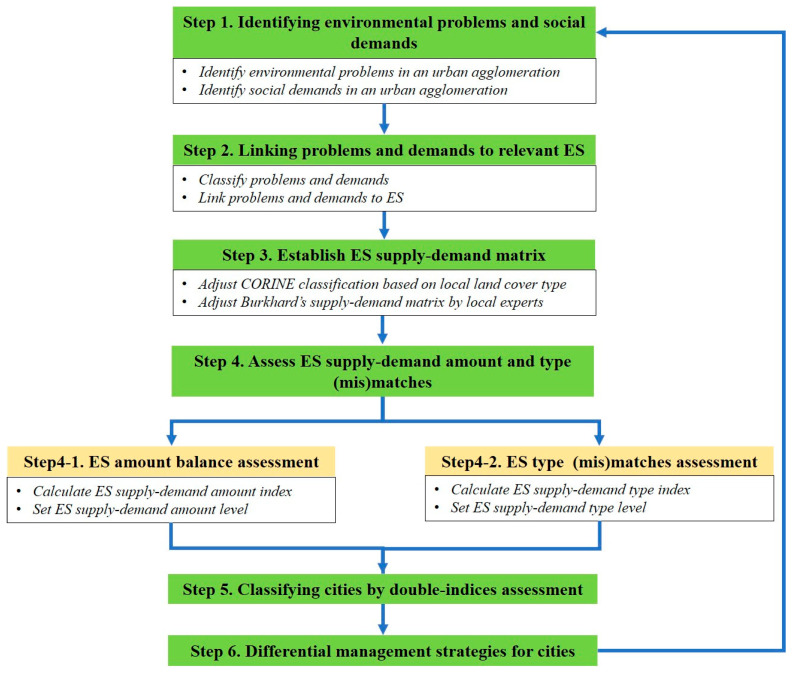
A general ES framework with six core steps for differential city management.

Differential city management based on ES supply–demand (mis)match assessment is a complex process in an urban agglomeration. Therefore, we present a general ES framework (Figure 1) for differential city management in an urban agglomeration. This framework comprises six core steps:

### 2.1. Step 1. Identify Environmental Problems and Social Demands

Identify environmental problems: regional environmental problems (water pollution, air pollution, soil erosion, flooding, and so on) may be identified through a review of government documents, academic reports, news, and combing with expert consultation and field works in an urban agglomeration.

Identify social demands: the demands from the society include materials, e.g., food and spirit, or aesthetics, which can be analyzed by the socio-economic development plan, social media reports, and interviews or surveys in an urban agglomeration.

### 2.2. Step 2. Link Problems and Demands to Relevant Ecosystem Services

Classify problems and demands: environmental problems can be classified based on natural process analysis, e.g., flooding related to the water flow process [39], and human activities analysis, e.g., the air pollution related to pollution emission [40]. Social demands can be classified into two categories: (1) demands for materials, e.g., crops, fresh air, clean water, timber, aquaculture, and (2) demands for spirit, e.g., recreation, tourism, aesthetic, cultural heritage.

Link problems and demands to ES: Based on supply–demand coupling analysis, the causal relationship between environmental problems and Regulating Services (RS) can be identified since RS (e.g., air quality regulation, water flow regulation, climate regulation) are closely related to ecological and environmental process [41,42,43]. Material demands are related to Provisioning Services (PS) [44] since PS was defined as final ecosystem goods to society, e.g., crops provision, freshwater provision. Spiritual demands are related to Cultural Services (CS) [45,46] since CS are closely related to spiritual perception.

### 2.3. Step 3. Establish Ecosystem Services Supply–Demand Matrix

The CORINE (Co-ordinated Information on the Environment) land cover classification based on European context provides a good reference for the land cover classification of other continents [47]. Land cover types in the CORINE classification can be transferred to natural or human-modified ecosystem types that provide or consume ecosystem goods and services [10,11,47]. In addition, the CORINE classification should be adjusted according to local land cover types [47].

Burkhard’s method constructs an ES matrix combining land cover information in the assessment of ecosystems’ capacities to supply ES and demand [10,11]. In the ES supply matrix, natural land cover types (e.g., forest, wetland) are given high capacities scores to provide several ES. In contrast, human-modified land cover types (e.g., urban fabric, industrial, or commercial areas) are given very low or no relevant capacities scores to provide RS and PS. Semi-natural land cover types (e.g., croplands) are often provided high capacities scores in specific PS. In the ES demand matrix, demands scoring for ES were suggested based on population numbers and average consumption patterns but also on land use activities and on their demands for certain services [10,15,48]. Human-modification land cover types with high population numbers and high human activities had high demands for multiple ecosystem services. Natural and semi-nature land cover types had low or no relevant demands for ES because normally fewer people were present there.

### 2.4. Step 4. Assess ES Supply–Demand Amount and Type (Mis)Matches

#### 2.4.1. Step 4-1. Assess City ES Supply–Demand (Mis)Matches Based on the ES Amount Index

According to previous studies of ES supply–demand index [15,31], we defined the ES Supply–demand Amount Index (ESAI) [15,31], which links the actual supply of the ecosystem with the demand of human beings, was employed to reveal ES supply–demand balance of single ES for each pixel in this study (Equation (1)):

(1) $$\mathrm{ESAI}=\frac{\left(S\;-\;D\right)}{\left(\mathrm{Smax}\;+\;\mathrm{Dmax}\right)/2\;}\;$$

where S and D, respectively, refer to specific actual supply and demand; Smax and Dmax, respectively, represent the maximum supply and human demand of specific ES extracted from the corresponding S and D space layers. This study provided the explanation that ESAI indicates the ES supply–demand status based on studies of [15,31]:

ESAI > 0 indicates that the ES supply meets the demand, i.e., there is surplus;

ESAI = 0, indicating ES supply and demand balance;

ESAI < 0 indicates that supply does not meet demand, i.e., there is a deficit.

Mean of amount index: *Mean* was employed to calculate the balance of multiple ES [15,31]. It is calculated as the arithmetic mean of ESAI of each category of ES

(2) $$Mean=\frac{1}{n}\overset{n}{\underset{i=1}{\sum }}\mathrm{ESARi}\;$$

where *n* is the number of estimated ES and *Mean* is the supply–demand index for each ES type.

Sum of amount index: *Sum* was defined to calculate the sum of the total amount of ESAI of each administrative unit (Equation (3)):

(3) $$Sum=\sum_{1}^{n}(Sn-Dn)*j\;$$

where *Sn* and *Dn*, respectively, refer to the specific actual supply and demand of each ES, *n* refers to the number of ES, *j* refers to the number of pixels in each administrative unit. It was then possible to calculate indices by coding the index using the ‘spatial calculation’ module in ArcGIS.

#### 2.4.2. Step 4-2. Assess City ES Supply–Demand (Mis)Matches Based on the ES Type Index

Previous studies, e.g., ES supply–demand budget [11], modified ES supply–demand balance index [36] and arithmetic mean of it [15,31] and made the comparison of the amount of ES supply and demand balance [12].

Based on previous studies, we addressed the importance of matches in various types of multiple ES supply and demand for urban land use management and introduced the ES Supply–demand Type Index (ESTI) for evaluating (mis)matches of ES supply–demand types.

(4) $$\mathrm{ESTI}=\frac{\left[N\left(\mathrm{surpuls}\right)+N\left(\mathrm{balance}\right)\right]-N\left(\mathrm{deficit}\right)}{N\left(\mathrm{surplus},\;\mathrm{balance},\;\mathrm{deficit}\right)\;}\;$$

Among them, N (surplus) indicates the number of ES that ESAI > 0; N (balance) indicates the number of ES that ESAI = 0; N (deficit) indicates the number of ES that ESDB < 0. N (total) represents the total number ES that ESAI > 0/= 0/< 0. This study gave the explanation of ESTI based on [15,31]:

If ESTI > 0, in the total number of ES, the number of highly matched (positive) and matched (zero) ESs is more than the number of imbalanced (negative). The higher the value, the higher the ES supply–demand matches from the perspective of the number of ES.

If ESTI = 0, in the total number of ES, the number of highly matched (positive) and matched (zero) ES is equal to the number of imbalanced (negative), indicating that the supply and demand match exactly.

If ESTI < 0, in the total number of ES, the number of highly matched (positive) and matched (zero) ES is less than the number of imbalanced (negative). The lower the value is, the lower the ES supply–demand from the perspective of the number of ES.

#### 2.4.3. Step 4-3. Rating ES Supply–Demand (Mis)Matches

We classified five levels of multiple ES supply–demand balance of each administrative unit according to amount matches (mean and sum) and type matches (Table 1).

Burkhard et al. 2014 suggested using equal intervals to classify ES supply, demand, and supply–demand matches [10]. In the first step, we used an equal interval of 0.5 to define different levels for ES supply–demand matches. Then, we defined −0.1~0.1 as an interval of the match since local experts considered that it was nearly impossible that ES supply was exactly equal to demand in the real world. The details of the five levels of multiple ES are presented in Table 1.

Thus, five levels of quantity and type in multiple ES were divided: I indicates that the ES supply highly exceeds demand (high match); II indicates that the ES supply exceeds demand (medium match); III indicates that ES supply is relatively equal to demand (match); IV indicates that ES does not meet demand (medium mismatch); V indicates that ES demand highly exceeds supply (high mismatch).

### 2.5. Step 5. Classify Cities by Double-Indices Assessment

**Figure 2:**
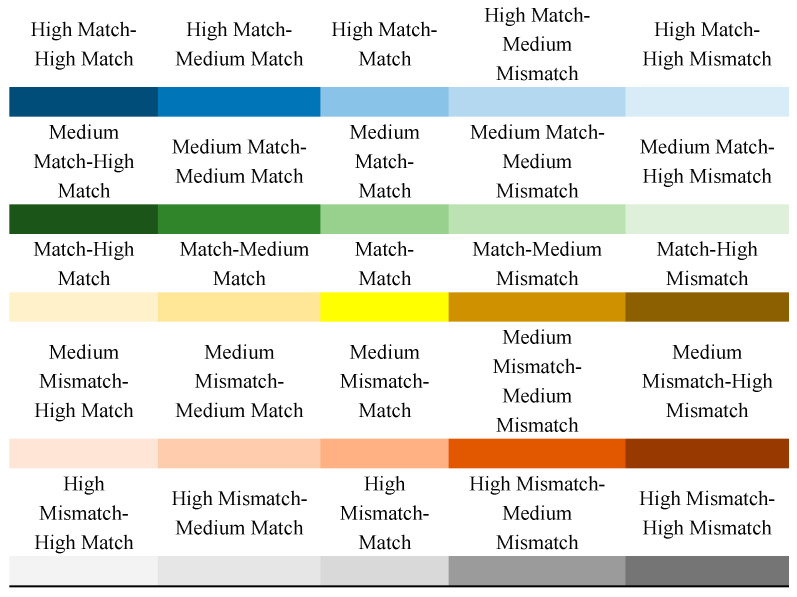
5 × 5 City Types of ES Supply–demand Amount Index (ESAI) and ES Supply–demand Match Type Index (ESTI).

Cities can be classified by double-indices assessment of amount matches and type matches (Figure 1). Based on five different levels of ESAI and ESTI, twenty-five (5 × 5) potential types can be defined totally (Figure 2), i.e., medium mismatch and medium match of ESAI and ESTI.

Sustainable land use management strategies can be put forward to each of the types. Unique types can be identified according to the (mis)matches of amount and type indicators in the specific study area.

### 2.6. Step 6. Design Differential Land Use Management Strategies

Differential land use management can be developed based on the double-indices (mis)matches assessment for three kinds of situations:

(1) For cities with both ‘match’ in ES amount and type, the ecosystem conservation policy, e.g., Ecological Redland policy, may be suggested for multiple ES surplus conservation, especially for cities with ‘high-match, high-match’.

(2) For cities with both ‘mismatch’ in ES amount and type, urban land control, population control, resource restraint policy for demand restraint may be the main city management policy.

(3) For cities with one ‘match’ and one ‘mismatch’, in ES amount and type, controlling the total amount of ES or making more matches of each main type of ES may be considered.

## 3. Case Study

### 3.1. Identify Environmental Problems and Demands

#### 3.1.1. Study Area

The YRDUA accounts for 11.7% of the national population with 2.14% of the total land area of China, and its output accounts for about 20% of total GDP [49,50]. It is one of the regions with the largest economic contribution in China and the highest intensity of human development activities, consumption of various resources and energy, and emission intensity of pollutants. Prior to the release of the Development Plan of the YRDUA (2016–2030), provinces and cities coped with environmental management independently with prominent overlapping, conflicts, and contradictions among the plans. Although the provinces and cities hold a highly accepted attitude towards the integrated supply of ecological products and ES and the integrated layout of environmental infrastructure construction in the YRDUA, it is difficult to achieve in-depth cooperation in actual actions due to the lack of scientific guidance, which restricts the coordinated development of ecological environmental governance and ecological security maintenance in the process of regional urbanization [50].

With the continuous evolution of regional urbanization, a series of ecological and environmental problems have aroused common concern, e.g., air pollution, water pollution [51]. Moreover, urban construction land has occupied many wetlands and arable lands in the recent ten years. Impervious surface area increase leads to the coastal city of waterlogging, surface runoff pollution, urban heat island effect intensifies, and serious eutrophication of Taihu Lake Basin [38].

**Figure 3:**
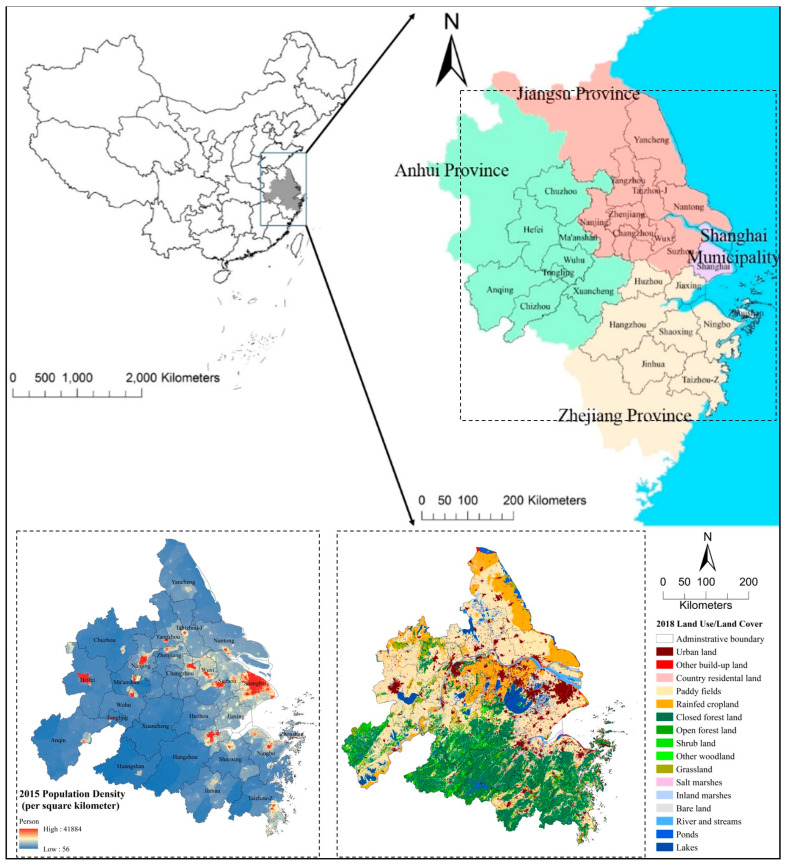
Location, Population Density (**Left below**), and Land Use (**Right below**) of Yangtze River Delta Urban Agglomeration.

The YRDUA is located in the lower reaches of the Yangtze River in Eastern China (Figure 3). The northern areas of the YRDUA were occupied by plain areas, while the southern areas were dominated by hills and mountains. The region is mainly located in the subtropical monsoon climate zone with relatively high annual mean temperature (13~18 °C) and abundant precipitation (776–2000 mm). The YRDUA covers approximately 206,000 km^2^, with approximately 147.4 million inhabitants.

In Figure 3 (Right below), there was large spatial heterogeneity in the distribution of land use/land cover in the YRDUA. Paddy fields and rainfed cropland are mainly distributed in the middle and northern plain areas, which occupied 34.69% and 11.38% of the total area. In contrast, closed forest land covered most of the southern mountainous areas, which occupied 23.89% of the region. In addition, urban land and country residential land are concentrated in the middle areas around Taihu Lake and scattered in the northern and southern plain areas. These two types totally accounted for 12.11% of the total.

Figure 3 (Left below) showed the spatial pattern of the population density of the study area in 2015. High-population-density areas were densely distributed in the middle and eastern areas of the YRDUA. These areas are mainly distributed along with the riverside of the Yangtze River and in the plain area of the Yangtze River Delta. They were mainly located in northern cities, e.g., Shanghai municipality, Suzhou, Wuxi, Changzhou, Yangzhou, Zhenjiang, Nanjing, and Taizhou-J in Jiangsu Province, Heifei and Ma’anshan in Anhui Province, and southern cities, e.g., Hangzhou, Shaoxing, Ningbo and southern areas in Taizhou-Z in Zhejiang Provinces.

There are twenty-five prefecture-level cities and one municipality in the YRDUA, comprising nine cities in Jiangsu Province, eight cities in Zhejiang Province, eight cities in Anhui Province, and Shanghai Municipality (Figure 3). These twenty-six cities were coding by (A1) Hefei, (A2) Anqing, (A3) Chizhou, (A4) Chuzhou, (A5) Ma’anshan, (A6) Tongling, (A7) Wuhu, (A8) Xuancheng in Anhui Province; (J1) Nanjing, (J2) Changzhou, (J3) Nantong, (J4) Suzhou, (J5) Taizhou-J, (J6) Wuxi, (J7)Yancheng, (J8) Yangzhou, (J9) Zhenjiang in Jiangsu Province; (S1) Shanghai Municipality; (Z1) Hangzhou, (Z2) Huzhou, (Z3) Jiaxing, (Z4) Jinhua, (Z5) Ningbo, (Z6) Shaoxing, (Z7) Taizhou-Z, and (Z8) Zhoushan.

#### 3.1.2. Identify Environmental Problems and Demands

Identify environmental problems: major environmental problems (flooding [52], water shortage [53], air pollution [15], greenhouse effect [54]) were identified for the YRDUA through government documents, environmental reports, and a review of the academic literature (Table 2): two national-level regional plan, one environmental report related to natural disasters and risk assessment, six watershed-level environmental reports related to flooding problems, a watershed-level environmental report related to water pollution problems, one municipal environmental report related to natural resource endowments, environmental conditions, and a literature review (e.g., [26,27,28,29,30]).

Identify social demands: huge population number and high economic development has generated high demands for comprehensive development in cities of the YRDUA [1,37]. High population generated high material demands, e.g., high demands for food and fresh water. Some megacities, such as Shanghai Municipality with over 20 million people [55], needed to transfer material resources (food, water) from other cities to meet local demands. High economic development required high material consumption in industrial cities [56], e.g., high consumption of industrial water resources. In the meantime, spiritual demands are generated with increasing living standards, such as tourism, recreation, and aesthetics. Demands for recreation in urban green parks and tourism in national parks were generated in cities that promote the communication of tourism among cities [57], such as Hangzhou (ecological tourism destination) with cities around [58].

**Table 2:** Key Government Documents/Environmental Reports and Related Ecosystem Services (After [51]).

Year	Level	Official Document	Social Demands	Related Ecosystem Services	Reference
2016	National	Development Plan of the Yangtze River Delta Urban Agglomeration (2016–2030)	Ecological/Environmental Integration; Resource Utilization; Cultural Integration	Regulating Services, e.g., Global Climate Regulation, Air Quality Regulation; Provisioning Services, e.g., Crops; Cultural Services, e.g., Recreation & Tourism, Knowledge System	[59]
2010	National	Regional Plan for the Yangtze River Delta Region (2009–2020)	Ecological/Environmental Integration; Resource Utilization; Cultural Integration	Regulating Services, e.g., Air Quality Regulation; Provisioning Services, e.g., Freshwater; Cultural Services, e.g., Recreation & Tourism	[60]
2014	Regional	Comprehensive Ecological Risk Prevention: Natural Disaster Factors and Risk Assessment in the Yangtze River Delta Region	Environmental Problem Solving: Global Warming; Green Effect; Flooding	Global Climate Regulation; Local Climate Regulation; Water Flow Regulation, Natural Hazard Regulation	[61]
2008–2017	Regional	The Health Status Report of Taihu Lake	Environmental Problem Solving: Flooding; Water and Soil Loss; Water Pollution	Water Purification, Freshwater, Aquaculture	[62]
2013–2018	Regional	Annual Report of Flood Control and Typhoon Prevention in Taihu Lake Basin	Environmental Problem Solving: Flooding; Water and Soil Loss	Water Flow Regulation, Natural Hazard Regulation, Erosion Regulation	[63]
2020	Municipal	Annual Report on the Resources and Environment of Shanghai	Environmental Problem Solving: Air Pollution; Water Pollution	Air Quality Regulation; Water Purification, Freshwater	[50]

### 3.2. Link Problems and Demands to Relevant Ecosystem Services

Link problems to RS: urban sprawl caused a massive amount of fragile ecosystem and the important ecological space crowding or damaged [64,65], leading to degradation of ecosystems and reducing supply to the multiple functions of the ecosystem [66], causing spatial mismatches between the supply and demand of multiple ES and resulted in environmental problems, such as flooding [52], water shortage [53], air pollution [15], greenhouse effect [54], etc. Based on the identification of environmental problems and their causal relationship to ES, eight RS targeted to regional and urban environmental problems were identified by local experts in the YRDUA (Table 2): global climate regulation, local climate regulation, air quality regulation, water flow regulation, water purification, erosion regulation, natural hazard regulation, and pollination.

Link material demands to PS: in some cases, forces on the demand side of economic growth [67] or population growth [68] are more influential than forces on the supply side. A highly agglomerated population generates acute social and economic activities, applies too much pressure on the ecological environment in the regions, and may also pose certain threats for social stability [69,70]. The population density in the Yangtze River Delta metropolitan region shows an overall characteristic of being higher in the north and lower in the south; that is, Shanghai had the highest population density, and the eight cities in Jiangsu Province have a higher population density than the six cities in Zhejiang Province [69,70]. High population density always means high demand for the total amount of different types of multiple ES supply in the YRDUA [12,15,36]. Based on high material demands and consumption generated by huge population numbers and high economic development in the YRDUA, six PS were identified by local experts: crops, biomass for energy, livestock (domestic), timber, aquaculture, and freshwater.

Link spiritual demands to CS: For CS (such as recreation and tourism), the carrying capacity and accessibility of urban green space is an important factor in the matching of supply and demand [15,35]. Based on spiritual demands for comprehensive development (Table 2), four CS were identified by local experts: recreation and tourism, landscape aesthetics and inspiration, knowledge systems, and cultural heritage and cultural diversity.

### 3.3. Establish Ecosystem Services Supply–Demand Matrix

In the study, we employed national land use datasets for classification of land use/land cover (LULC) in the YRDUA in 2018, which were produced by the Institute of Remote Sensing and Digital Earth, Chinese Academy of Sciences through interpretation of the Landsat TM or ETM images at a 30 m resolution. The overall accuracy of classification with ground-based survey data was over 85.35%.

Firstly, we corresponded CORINE land cover system with a local land cover system with local expert knowledge for establishment of ES supply–demand land cover matrix (Table 3). There are sixteen different land cover classes: (1) urban land, (2) country residential land, (3) other built-up land, (4) paddy fields, (5) rainfed croplands, (6) forest land, (7) open forest land, (8) other forest land (orchards), (9) shrub land, (10) grassland, (11) bare land, (12) inland marshes, (13) salt marshes, (14) lakes, (15) ponds, and (16) river and streams.

Secondly, we interviewed twenty local experts for ES assessment, eight from government agencies (two from Shanghai, two from Jiangsu, two from Zhejiang, two from Anhui Province), three from East China Normal University, one from Nanjing University, three from Anhui Normal University, one from Jiangsu University, two from an NGO in Zhejiang Province, and a green enterprise in Jiangsu. All experts are familiar with the YRDUA.

Thirdly, we established the ES supply–demand matrix by relating the sixteen land cover classes with the eighteen ES for the YRDUA (Table 4 and Table 5). In the first step, we assigned a score for each matrix sheet simply by applying the original matrix presented by Burkhard, Kandziora, Hou, and Müller [10]. Although the original score matrix focused on the European context, it provides a good reference for China. Land cover types in Burkhard’s European studies could correspond to China’s local land covers types that provided comparable ES supply capacities [36,47,71], though these capacities were modified by local expert’s knowledge according to the local socio-ecological background (e.g., vegetation, terrain, hydrology) [6,47,51]. For estimating the ES demands, RS and PS estimation were based on local population density and average consumption patterns but also on land use activities and on their demands for certain services [6,15,35,51]. For instance, population density above 20,000 people per kilometers showed a very high demand in multiple RS and PS mainly distributed in the urban land in cities, e.g., Shanghai Municipality and Nanjing in Province (Figure 3). For CS estimation, a multiplication of the population density and the local government guidance on green space provision per capita during the study period were applied for scores modification [12,35]. For example, 60 m^2^ per person of ecological land (forest lands, wetlands) suggested in local studies were considered by local experts in estimation [72].

Then, we divided twenty experts into four groups according to their locations, with five experts in each group: Shanghai group, Zhejiang group, Jiangsu group, and Anhui Group. We calculated the ‘Median’ scores of experts in each group as the final score for each ES to each land cover [51].

### 3.4. Assess ES Supply–Demand Amount and Type (Mis)Matches

**Figure 4:**
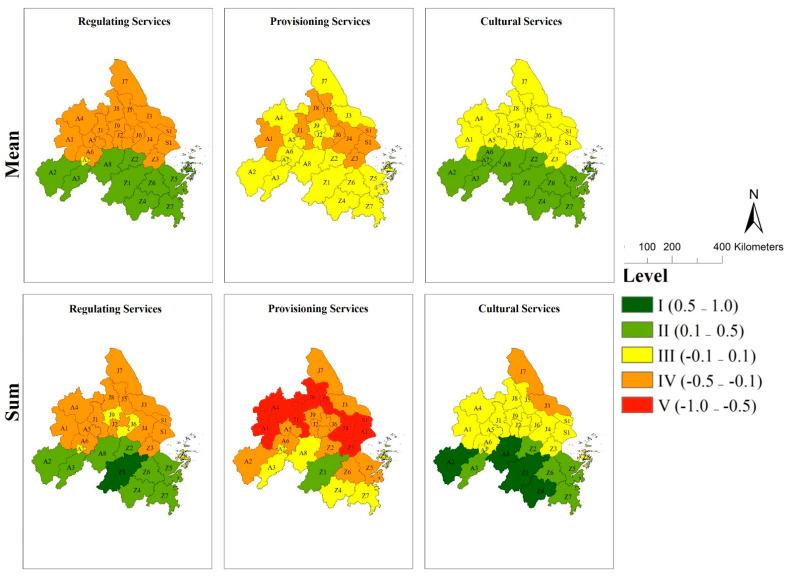
Spatial Pattern and Levels of Mean and Sum of Regulating Services, Provisioning Services and Cultural Services: (A1) Hefei, (A2) Anqing, (A3) Chizhou, (A4) Chuzhou, (A5) Ma’anshan, (A6) Tongling, (A7) Wuhu, (A8) Xuancheng in Anhui Province; (J1) Nanjing, (J2) Changzhou, (J3) Nantong, (J4) Suzhou, (J5) Taizhou-J, (J6) Wuxi, (J7)Yancheng, (J8) Yangzhou, (J9) Zhenjiang in Jiangsu Province; (S1) Shanghai Municipality; (Z1) Hangzhou, (Z2) Huzhou, (Z3) Jiaxing, (Z4) Jinhua, (Z5) Ningbo, (Z6) Shaoxing, (Z7) Taizhou-Z, and (Z8) Zhoushan.

**Figure 5:**
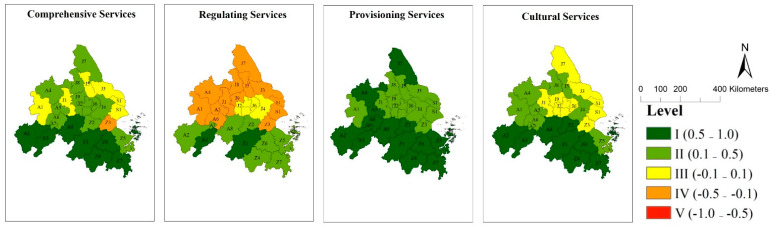
Spatial Pattern and Levels of Type of Comprehensive Services, Regulating Services, Provisioning Services and Cultural Services: (A1) Hefei, (A2) Anqing, (A3) Chizhou, (A4) Chuzhou, (A5) Ma’anshan, (A6) Tongling, (A7) Wuhu, (A8) Xuancheng in Anhui Province; (J1) Nanjing, (J2) Changzhou, (J3) Nantong, (J4) Suzhou, (J5) Taizhou-J, (J6) Wuxi, (J7)Yancheng, (J8) Yangzhou, (J9) Zhenjiang in Jiangsu Province; (S1) Shanghai Municipality; (Z1) Hangzhou, (Z2) Huzhou, (Z3) Jiaxing, (Z4) Jinhua, (Z5) Ningbo, (Z6) Shaoxing, (Z7) Taizhou-Z, and (Z8) Zhoushan.

Based on the double-indices method put forward in 2.4, ES supply–demand amount and type (mis)matches of twenty-six cities in the YRDUA were assessed. The results are as follows: (Figure 4 and Figure 5)

#### 3.4.1. Assess City ES Supply–Demand (Mis)Matches Based on the ES Amount Index

The results of the mean index assessment were: (1) ten surplus cities which were medium surplus (e.g., A2, Z1, Z2, Z4, etc.), one balance city (A7), and fifteen deficit cities which were medium deficit (e.g., A1, J1, S1, Z3, etc.) in RS; (2) no surplus cities and eighteen match cities (e.g., A2, J2, Z1, Z4, etc.) and eight deficit cities (e.g., A1, J1, S1, Z3, etc.) in PS; (3) twelve surplus cities which were medium surplus (e.g., A2, Z1, Z2, Z4, etc.), fourteen match cities (e.g., A1, J1, S1, Z3, etc.) in CS.

The results of the sum index assessment were: (1) one high surplus city (Z1), and eight medium surplus cities (e.g., A2, Z2, Z4, Z5, etc.), four match cities (A7, J6, J9, Z8), and thirteen medium deficit cities (e.g., A1, J1, S1, Z3, etc.) in RS; (2) one surplus city (Z1), six match cities (e.g., A3, Z4, Z7, etc.), eleven medium deficit cities (e.g., A2, J2, J6, Z5, etc.), and eight high deficit cities (e.g., A1, J1, S1, Z3, etc.) in PS; (3) four high surplus cities (A2, A8, Z1, Z4), five medium surplus cities (A3, Z2, Z5–Z7), fifteen match cities (e.g., A1, J1, S1, Z3, etc.), and two medium deficit cites (J3, J7) in CS.

We defined fourteen cities (S1, J1–J9, A1, A4–A5, Z3) as southern cities and twelve cities (Z1–Z2, Z4–Z8, A2–A3, A6–A8) as northern cities of the region according to their locations. In general, twenty-six cities displayed a similar spatial pattern in mean and sum (mis)match index in RS, PS, and CS (Figure 4). However, the match levels of southern cities were mainly higher than northern cities in RS, PS, and CS.

#### 3.4.2. Assess City ES Supply–Demand (Mis)Matches Based on the Type Index

The results of type index assessment were: (1) eight high match cities (e.g., A3, Z1, Z4, Z6, etc.), twelve medium match cities (e.g., A4, A7, J4, Z2, etc.), five match cities (A1, J1, J3, J5, S1) and one medium mismatch city (Z3) in the Comprehensive Services; (2) two high match cities (A3, Z1), nine medium match cities (e.g., A7, Z2, Z4, Z6, etc.), three match cities (J2, J4, J6), and twelve medium mismatch cities (e.g., A1, J1, S1, Z3, etc.) in RS; (3) fifteen high match cities (e.g., A3, J7, Z1, Z6.) and eleven medium match cities (e.g., A1, J1, S1, Z3, etc.) in PS; (4) nine high match cities (e.g., A2, Z1, Z2, Z4, etc.), nine medium match cities(e.g., A4, A7, J4, J5, etc.), and eight match cities (e.g., J1, J6, S1, Z3, etc.) in CS.

In general, twenty-six cities displayed a similar spatial pattern in type (mis)matches index in the Comprehensive Services and CS (Figure 5). However, northern cities were Level IV and Level III, whereas southern cities were mainly Level II.

### 3.5. Classify Cities by Double-Indices Assessment

**Figure 6:**
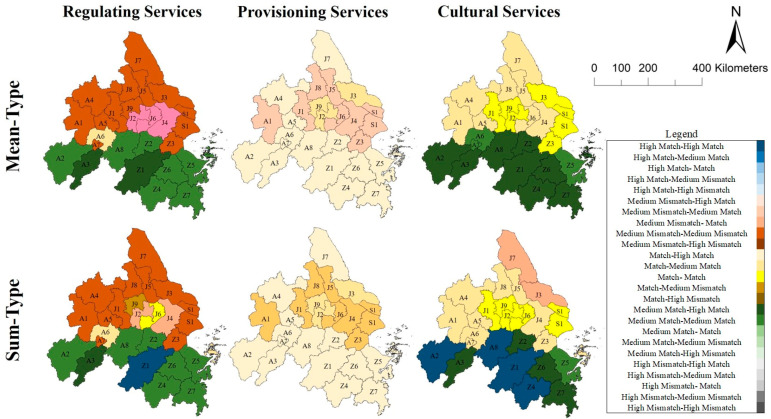
Double-indices (Mean-Type and Sum-Type) Spatial Pattern of Regulating Services, Provisioning Services, and Cultural Services: (A1) Hefei, (A2) Anqing, (A3) Chizhou, (A4) Chuzhou, (A5) Ma’anshan, (A6) Tongling, (A7) Wuhu, (A8) Xuancheng in Anhui Province; (J1) Nanjing, (J2) Changzhou, (J3) Nantong, (J4) Suzhou, (J5) Taizhou-J, (J6) Wuxi, (J7)Yancheng, (J8) Yangzhou, (J9) Zhenjiang in Jiangsu Province; (S1) Shanghai Municipality; (Z1) Hangzhou, (Z2) Huzhou, (Z3) Jiaxing, (Z4) Jinhua, (Z5) Ningbo, (Z6) Shaoxing, (Z7) Taizhou-Z, and (Z8) Zhoushan.

The results integrating ES amount and type (mis)matches assessment in twenty-six cities are as follows:

Mean-Type (Figure 6): (1) for RS, twenty-six cities can be classified into five kinds of (mis)match cities: two cities (A3 and Z1) were ‘medium match, high match’, eight cities (e.g., A2, Z2, Z4 and Z6) were ‘medium match, medium match’, one city (A7) was ‘match, medium match’, three cities (J2, J4, J6) were ‘medium mismatch, match’ and twelve cities (e.g., A1, J1, S1 and Z3) were ‘medium mismatch, medium mismatch’; (2) for PS, three kinds of (mis)match cities: fifteen cities (e.g., A2, A3, Z1, and Z2) were ‘match, high match’, three cities (J2, J3, J9) were ‘match, medium match’, and eight cities (e.g., A1, J1, S1, and Z3) were ‘medium mismatch (IV), medium match’; (3) For CS, four kinds of (mis)match cities: nine cities (e.g., A2, Z1, Z2, and Z4) were ‘medium match, high match’, three cities (A6, A7, Z5) were ‘medium match (II), medium match’, six cities (A1, A4, J4, J5) were ‘match, medium match’, and eight cities (e.g., J1, J2, S1, and Z3) were ‘match, match’.

Sum-Type (Figure 6): (1) for RS, twenty-six cities can be classified into eight kinds of (mis)match cities: one city (Z1) was ‘high match, high match’, one city (A3) was ‘medium match, high match’, seven cities (e.g., A2, Z2, Z4 and Z6) were ‘medium match, medium match’, two cities (A7, Z8) were ‘balance or match, medium match’, one city (J6) was ‘match, match’, two cities (J2, J4) were ‘medium mismatch, match’, one city (J9) was ‘match, medium balance’, eleven cities (e.g., A1, J1, S1, and Z3) were ‘medium mismatch, medium mismatch’; (2) for PS, three kinds of (mis)match cities: fifteen cities (e.g., A2, A3, Z1 and Z2) were ‘match, high match’, three cities (J2, J3, J9) were ‘match, medium match’, and eight cities (e.g., A1, J1, S1 and Z3) were ‘medium mismatch, medium match’; (3) for CS, six kinds of (mis)match cities: four cities (A2, A8, Z1, Z4) were ‘high match, high match’, four cities (A3, Z2, Z6, Z7) were ‘medium match, high match’, one city (Z5) was ‘medium match, medium match’, nine cities (e.g., A1, A4, J4, and J5) were ‘match, medium match’, six cities (e.g.,J1, J2, S1 and Z3) were ‘match, match’ and two cities (J3, J7) were ‘medium mismatch, match’. 

### 3.6. Design Differential Land Use Management Strategies

Based on the results of the double-indices assessment, four kinds of strategies were designed for cities in the YRDUA:

(1) For the cities matched in both amount and type (Figure 6)—‘medium match, high match’, ‘medium match, medium match’, and ‘match, medium match’ in RS, ‘match, high match’ in PS, and ‘medium match, high match’ in CS—ecosystem conservation policies, e.g., Ecological Redline Policy, should be the main measurement of these cities for RS, PS, and CS management. These cities can be potential multiple ES providing areas for cities with mismatches in both amount and type. For example, forest, wetland, and other natural ecosystem conservation should be strengthened in the transboundary areas of cities, and the implementation of joint construction and co-protection of land use management should be carried out.
(2) For the cities matched in amount but mismatched in type (Figure 6)—‘match, medium mismatch’ in RS, ‘match, medium mismatch’ in PS, and ‘match, medium mismatch’ and ‘match, mismatch’ in CS, i.e., these cities with total balance in ES but mismatches in multiple types of ES—it is suggested to carry out policies that can promote the synergy of multiple ES simultaneously. For example, these cities should carry out the PS supply capacity of cropland ecosystem by ‘Prime Farmland Policy’, as well as the ‘Grain for Green’ policy [38] for increasing the RS supply simultaneously, based on studies of ES multifunction management;
(3) For the cities mismatched in both amount and type (Figure 6)— ‘medium mismatch, medium mismatch’ in RS—it is suggested that urban sprawl and population control policy should be emphasized, especially for PS management, since reducing demands of ES may be more effective if multiple ES supply may be hard to meet the demand. External environmental cooperation among different-level cities should also be carried out, e.g., payment for ES in RS and PS management, and tourism cooperation in CS management, since the demand of multiple ES of these types of cities cannot be satisfied by ES supply itself;
(4) Cities mismatched in amount but matched in type (Figure 6)—‘medium mismatch, match’ in RS, ‘medium mismatch, medium match’ in PS, i.e., cities with a total imbalance in ES but matches in multiple types of ES—should focus on demand control strategies for specific ES.

Differential land use management for cities should be fundamental to the consideration of influences of different natural and anthropogenic factors in cities of an urban agglomeration. In the case study, the spatial mismatches between ES supply and demand in the cities of YRDUA were influenced by both natural and anthropogenic factors [36,37,47,73,74].

On the one hand, there was spatial heterogeneity in the distribution of ecosystems in the YRDUA. The forest ecosystems were the main sources of multiple ES supply, which were mainly distributed in the mountainous areas of southern and western cities with low disturbance of human activities, such as Hangzhou, Anqing. In contrast, the cropland and urban land were the main sources of multiple ES demand, which were mainly distributed in the plain areas of northern and eastern cities with high intensity of human disturbance, such as Shanghai, Suzhou. This was the natural reason that ES supply–demand (mis)matches results showed high spatial autocorrelation that both matches in amount and type in the south and both in mismatches in the north.

On the other hand, concentrated distribution and a huge number of the urban and rural population in the northern and central cities, as well as the economic development, have generated high demand in both total amounts and each type of ES in the YRDUA [37,74]. High-intensity agriculture and urbanization in the north and the middle cities of YRDUA destroyed the integrity of regional and urban ecosystems [47], thus weaken ES supply and aggregated the mismatches of ES supply and demand in both total amount and each type of ES in those cities. Moreover, environmental pollution, e.g., air and water environmental pollutions, is also caused by the ES supply–demand mismatch in the north and the middle of the region. For example, the problem of water pollution in the Taihu Lake Basin brought water quality shortage to several cities in the YRDUA [75,76], resulting in mismatches in freshwater provision service and water purification service.

Under the co-effect of natural and anthropogenic factors, a variety of ES supply and demand (mis)match combinations are generated. The objectives of different city management are to manage the co-effect of natural and anthropogenic factors for different (mis)matches combination in different cities. A city that matched in both amount and type assessment, e.g., Hangzhou, should take action in forest ecosystem conservation since the distribution of forest was the main natural factor in ES supply–demand matches of this city. A city that is mismatched in both amount and type assessment, e.g., Shanghai, should control the urban growth and population number to restrict the demand since few natural ecosystems were distributed in the city, and high demand was generated by high population numbers. A city that matched in amount but mismatched in type means the number of ES matches types was fewer than the number of ES mismatches types. For specific ES types matches management, specific natural and anthropogenic factors should be found for specific land use management. A city that mismatched in amount and matched in types means that the overall amount of ES supply cannot match the total demand. Ecosystem conservation policy and population control should be performed simultaneously to make a positive co-effect of natural and anthropogenic factors.

## 4. Discussion

### 4.1. ES Framework

In previous studies, several ES frameworks were put forward for environmental management and decision support [10,11,47,51,77]. These ES frameworks have evolved with increasing requirements of environmental management. Earlier frameworks only focused on ES supply-side assessment but ignored the ES demand-side assessment; thus it can only be applied in regional ecosystem conservation management (e.g., ecological redline delineation) [47,78]. Then, some frameworks considered both ES supply and demand but did not link ES to LULC types that could not be applied in spatial planning and management [54,79]. Current frameworks considered not only both ES supply and demand but also the link to LULC. Recently, Cai et al. (2020) developed a general ES framework integrating Burkhard’s ES supply–demand budget with flow direction analysis, identifying ES city type and spatial relations. This framework could be applied in intercity environmental cooperation and payment for ES mechanisms in a highly urbanized region [51]. Based on previous studies, this study presented a general ES framework for differential city management in an urban agglomeration. This framework was designed for ES supply–demand (mis)match assessment of cities and classified cities based on the assessment for differential land use management in an urban agglomeration.

### 4.2. Double-Indices Assessment

The ES supply–demand amount (mis)match assessment was considered as an important criterion in environmental management at different scales [20,31]. For example, Li et al. (2016) developed an ES supply–demand amount index and addressed the idea of arithmetic mean of balance matches for comprehensive ecological function zone assessment. Lorilla, et al. [80] assessed the ES supply–demand balance matches for islands’ sustainable development. Chen et al. (2019) presented and employed the arithmetic mean of the ES supply–demand amount index in a case study of urban land use management in Shanghai Municipality. It is disappointing that these studies do not include the ES supply–demand type (mis)match assessment and ignores some useful information on different types of ES supply and demand for environmental management. This may lead to a biased or incomplete decision in environmental management. This study addressed the necessity of including both ES supply–demand type (mis)match assessment and the amount (mis)match assessment for comprehensive environmental management in an urban agglomeration. The double-indices assessment can provide more information for cities’ decision-makers for differential governance, such as different levels and spatial patterns of (mis)matches. In the case study, we found differences in cities with inconsistent matches levels in the double-indices assessment within the single-index assessment. For instance, compared with the single-index assessment, A7 of ‘match, medium match’ and (J2, J4, J6) of ‘medium mismatch, match’ were new groups for RS in the Mean-Type double-indices assessment. For another, (J2, J3, J9) of ‘match, medium match’ was a new city category for PS in the Sum-Type double-indices assessment compared with the single-index assessment.

### 4.3. Contributions and Limitations

In this study, we developed a general framework based on ES supply–demand (mis)match assessment for differential city management in an urban agglomeration. This framework not only made up for deficiencies in unified city management in socio-ecological integration of an urban agglomeration but also provide a new insight for environmental management at other scales. The aim of the double-indices assessment was to assess ES supply–demand (mis)matches in amount and type. Prior to the single-index assessment, the double-indices assessment can help to understand the ES supply–demand status comprehensively in the study area.

This study has limitations, as follows: The scoring of ES supply–demand by local expert knowledge has certain uncertainty [36,81], especially for scoring ES demands, and it was influenced by several factors, including data availability, population density, local income, age, etc. Therefore, the results of the study should be carefully applied in other areas and should be calibrated by local data.

## 5. Conclusions

In this study, we combined ES supply–demand amount (mis)match with type (mis)match to present a double-indices assessment method to have a deeper understanding of the ES supply–demand status comprehensively in a study area. A general ES framework with six core steps for differential city management in an urban agglomeration was presented and applied in the ES supply–demand (mis)match assessment of cities in the YRDUA. Twenty-six cities in the YRDUA were classified into five kinds of cities with different levels of ES supply–demand (mis)matches for RS, three kinds of cities for PS, and four kinds of cities for CS. Differential city management strategies were designed. Despite its limitations, this study can be a reference to provide insights into ES supply–demand (mis)match assessment and management.

## Figures and Tables

**Table 1:** Five level of amount index and type index.

Level	Meaning	Range
I	High Match	0.5 ≤ Amount/Type ≤ 1.0
II	Medium Match	0.1 ≤ Amount/Type < 0.5
III	Match	−0.1 < Amount/Type < 0.1
IV	Medium Mismatch	−0.5 < Amount/Type < −0.1
V	High Mismatch	−1.0 ≤ Amount/Type ≤ −0.5

**Table 3:** Relationship between CORINE and Yangtze River Delta Urban Agglomeration System.

CORINE Land Cover	YRDUA Land Cover	Ecosystem Types
Continuous urban fabric	Urban land	Urban
Discontinuous urban fabric	Country residential land	
Construction sites	Other built-up land	
Non-irrigated arable land	Rainfed croplands	Cropland
Permanently irrigated arable land	Paddy fields	
Broad-leaved forest	Forest land/Open Forest land/Other woodland	Woodland and forest
Coniferous forest		
Mixed forest		
Transitional woodland shrub	Shrub land	
Natural grassland	Grassland	Grassland
Bare rock	Bareland	Sparsely vegetated areas
Inland marshes	Inland marshes	Wetlands
Salt marshes	Salt marshes	
Water bodies	Lakes/Ponds	Rivers and lakes
Water courses	River and Streams	

**Table 4:** Ecosystem services supply matrix. Scale from 0- = no relevant flow; 1 = low relevant flow; 2 = relevant flow; 3- = medium relevant flow; 4- = high relevant flow; and 5- = very high (maximum) relevant flow (After Burkhard et al., 2014).

	Regulating Services	Global Climate Regulation	Local Climate Regulation	Air Quality Regulation	Water Flow Regulation	Water Purfication	Erosion Regulation	Natural Hazard Regulation	Pollination	Provisioning Services	Crops	Biomass of Energy	Livestock (Domestic)	Timber	Aquaculture	Freshwater	Culture Services	Recreation & Tourism	Landscape Aesthetics & Inspiration	Knowledge System	Cultural Heritage & Cultural Diversity
Paddy fields		0	2	1	4	0	0	0	1		4	1	0	0	0	0		1	1	1	2
Rainfed Cropland		1	2	1	2	0	0	1	3		4	4	0	0	0	0		1	1	1	1
Closed forest land		4	5	5	3	4	5	3	1		0	1	0	2	0	0		4	4	4	2
Shrub land		2	2	1	1	2	1	1	1		0	1	0	1	0	0		2	3	4	1
Open forest land		2	4	4	2	3	4	2	0		0	1	0	2	0	0		3	3	3	1
Other woodland		2	2	2	2	1	2	2	3		0	0	0	0	0	0		2	1	1	3
Grassland		2	2	0	1	3	5	1	2		0	0	2	0	0	0		3	4	4	2
River and Streams		0	1	0	3	3	0	3	0		0	2	0	0	3	3		4	4	3	2
Lakes		1	3	0	3	2	0	3	0		0	0	0	0	3	2		5	4	3	2
Ponds		0	2	0	2	1	0	1	0		0	0	1	0	4	0		1	1	1	1
Salt marshes		0	1	0	1	1	2	1	4		0	0	1	0	0	0		3	2	3	0
Inland marshes		2	2	0	2	2	1	4	1		0	0	1	0	0	0		1	2	3	1
Urban land		0	0	0	0	0	2	0	1		0	0	0	0	0	0		3	2	2	1
Contry residential land		0	0	0	0	0	1	0	2		0	0	0	0	0	0		2	1	2	2
Other build-up land		0	0	0	0	0	0	0	0		0	0	0	0	0	0		0	0	0	1
Bareland		0	0	0	0	1	1	0	0		0	0	0	0	0	0		2	3	2	1

**Table 5:** Ecosystem services demand matrix. Scale from 0- = no relevant demand; 1 = low relevant demand; 2 = relevant demand; 3- = medium relevant demand; 4- = high relevant demand; and 5- = very high (maximum) relevant demand (After Burkhard et al., 2014).

	Regulating Services	Global Climate Regulation	Local Climate Regulation	Air Quality Regulation	Water Flow Regulation	Water Purfication	Erosion Regulation	Natural Hazard Regulation	Pollination	Provisioning Services	Crops	Biomass of Energy	Livestock (Domestic)	Timber	Aquaculture	Freshwater	Culture Services	Recreation & Tourism	Landscape Aesthetics & Inspiration	Knowledge System	Cultural Heritage & Cultural Diversity
Paddy fields		2	2	1	5	5	2	2	2		0	1	0	0	0	5		0	0	2	3
Rainfed Cropland		2	2	1	2	0	3	2	3		0	1	0	1	0	0		2	1	2	3
Closed forest land		0	0	0	0	0	0	0	0		0	0	0	0	0	0		0	0	0	0
Shrub land		0	0	0	0	0	0	0	0		0	0	0	0	0	0		0	0	0	0
Open forest land		0	0	0	0	0	0	0	0		0	0	0	0	0	0		0	0	0	0
Other woodland		1	2	1	2	3	1	3	3		0	1	0	1	0	0		2	1	2	3
Grassland		0	0	0	0	0	0	0	0		0	0	0	0	0	0		0	0	0	0
River and Streams		0	0	0	0	0	0	0	0		0	0	0	0	0	0		0	0	0	0
Lakes		0	0	0	0	0	0	0	0		0	0	0	0	0	0		0	0	0	0
Ponds		0	0	0	0	0	0	0	0		0	0	0	0	0	0		0	0	0	0
Salt marshes		0	0	0	0	0	0	0	0		0	0	0	0	0	0		0	0	0	0
Inland marshes		0	0	0	0	0	0	0	0		0	0	0	0	0	0		0	0	0	0
Urban land		4	5	5	4	5	1	5	1		5	5	5	3	5	5		4	4	3	4
Contry residential land		3	5	5	5	4	1	4	2		4	4	4	3	4	5		4	4	3	2
Other build-up land		1	2	1	2	2	2	3	0		0	4	0	4	0	2		0	0	0	0
Bareland		0	0	0	0	0	0	0	0		0	0	0	0	0	0		0	0	0	0

## Data Availability

Data are contained within the article.
